# Supplementary material for: Observation of giant spin-split Fermi-arc with maximal Chern number in the chiral topological semimetal PtGa
Source: Nat Commun. 2020 Apr 27;11:2033. doi: 10.1038/s41467-020-15865-x (PMC7184610; doi:10.1038/s41467-020-15865-x)
Supplement: Supplementary file 1 — Supplementary Information [file 41467_2020_15865_MOESM1_ESM.pdf]

SUPPLEMENTARY INFORMATION

Yao *et al.* Observation of giant spin-split Fermi-arc with maximal Chern number in the chiral topological semimetal PtGa

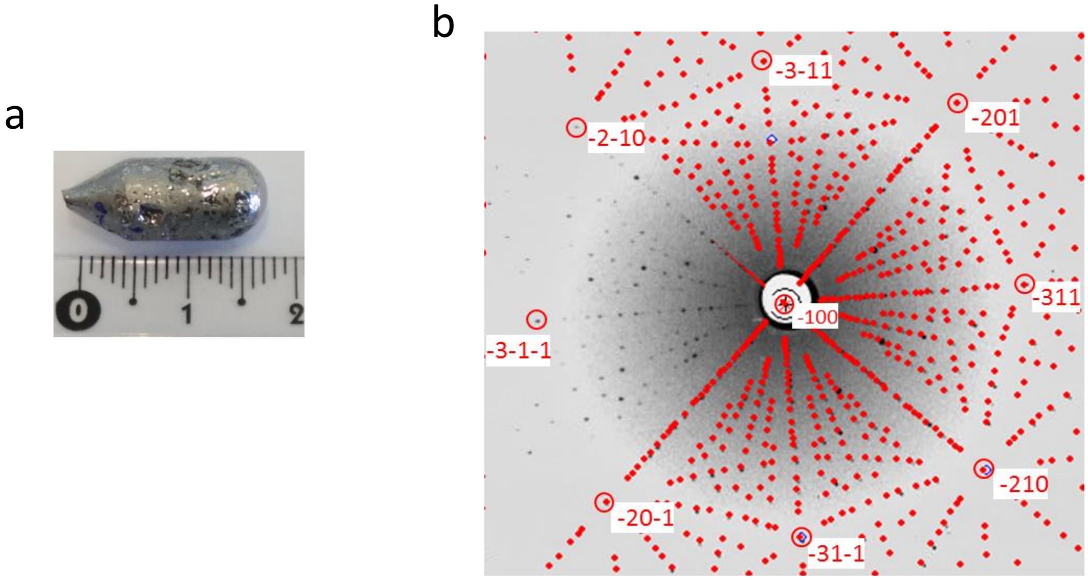

Supplementary Figure 1 |Optical image and X-ray diffraction of crystal (a) A optical picture of the as grown PtGa single crystal; (b) Representative Laue diffraction pattern of the [100] oriented PtGa single crystal superposed with the simulated pattern confirming high crystal quality.

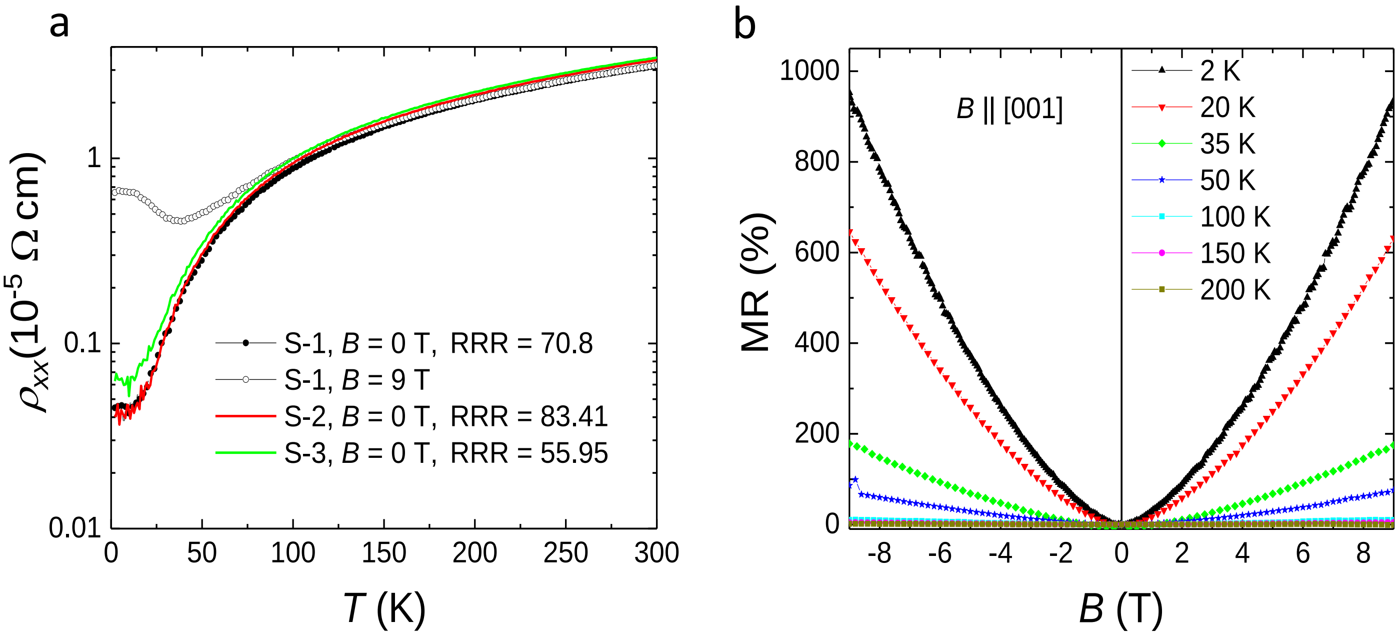

Supplementary Figure 2 | Resistivity of PtGa. (a) Temperature dependence of the longitudinal resistivity with current applied along the [100] direction of various batches of PtGa single crystals. For sample S-1, resistivity data with 9 T field applied along [001] are shown as well; (b) Giant magnetoresistance, [$MR = [\rho(B) - \rho(0)] / \rho(0)$] observed for sample S-1 at various temperatures.

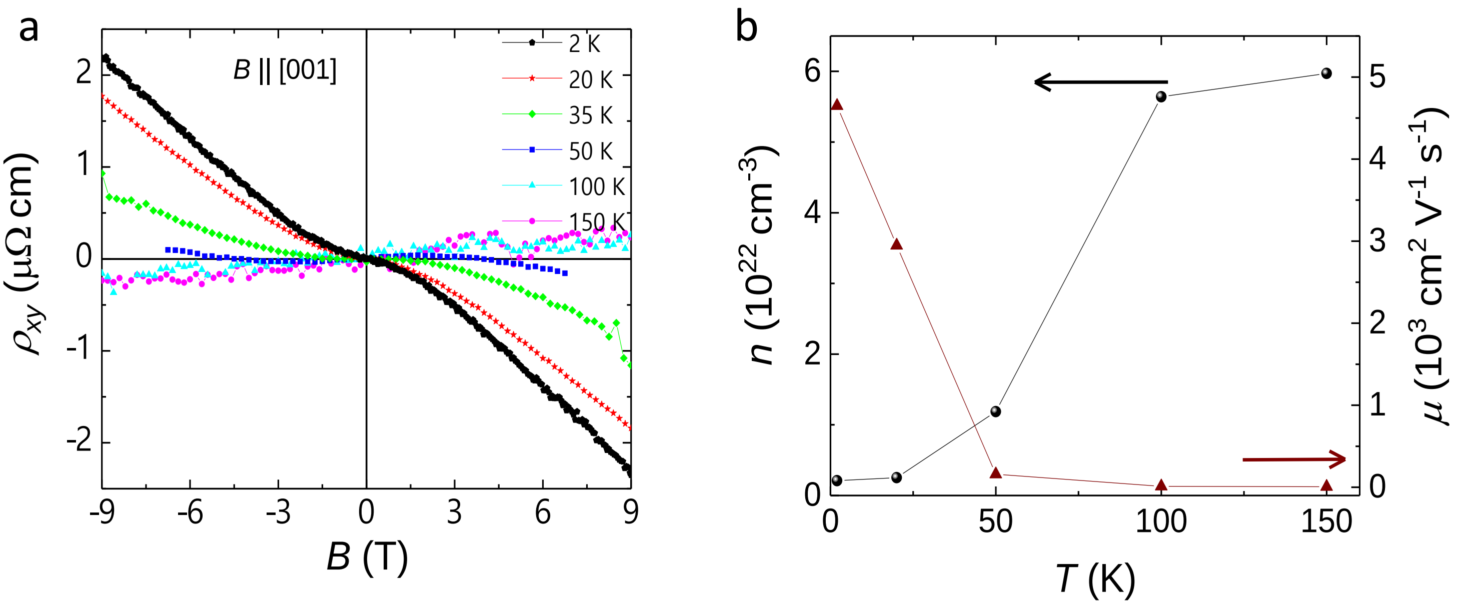

Supplementary Figure 3 | Hall characteristics of PtGa. (a) Magnetic-field dependence of Hall resistivity for sample S-1; (b) Carrier concentration and mobility estimated from high-field slope at various temperatures. For simplicity, a single-carrier Drude band model was used to estimate the charge-carrier density (n) and mobility (μ) using $n(T) = 1/[eR_H(T)]$ and $\mu(T) = R_H(T)/\rho_{xx}(T)$, where e denotes the electronic charge and R_H the Hall coefficient.

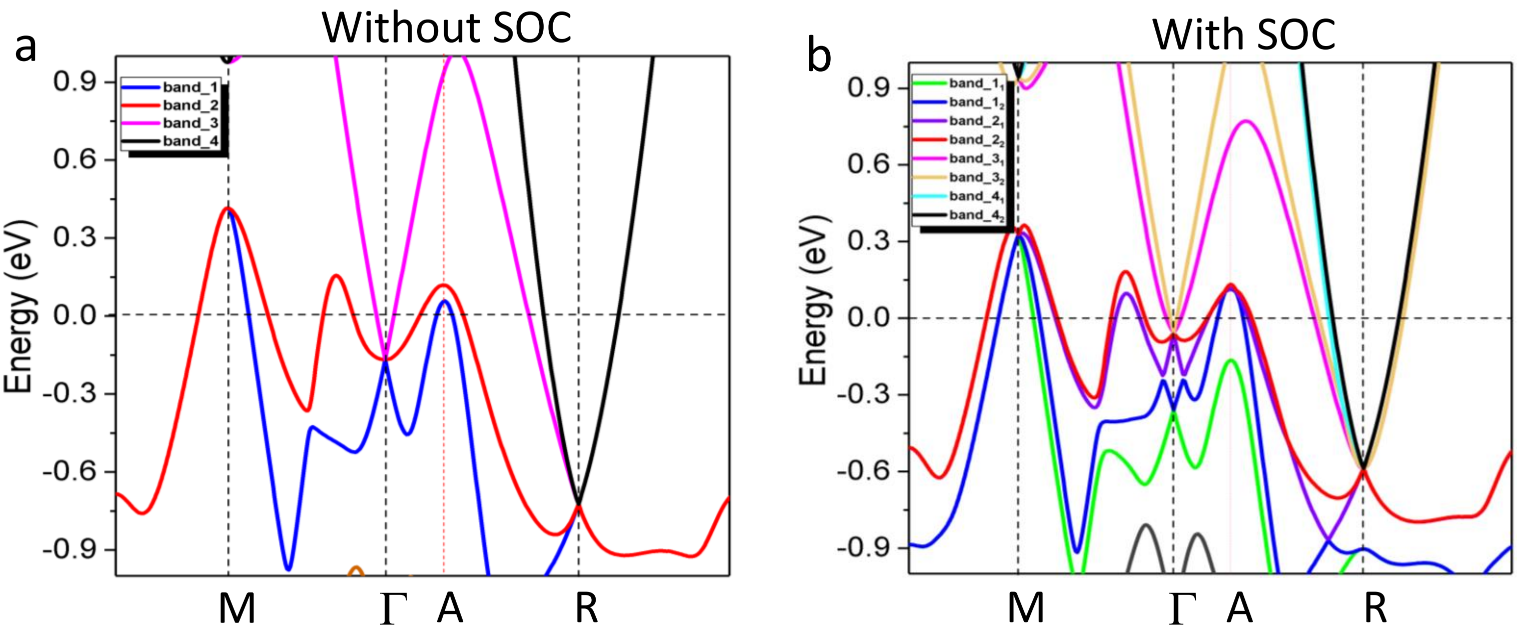

Supplementary Figure 4 | Detailed band structure of PtGa without (a) and with (b) incorporation of spin-orbit coupling. Corresponding spin-split bands are highlighted with respective colors. There are three hole pockets along the high symmetry line of Γ-R. For convenient, we denote the center as point A.

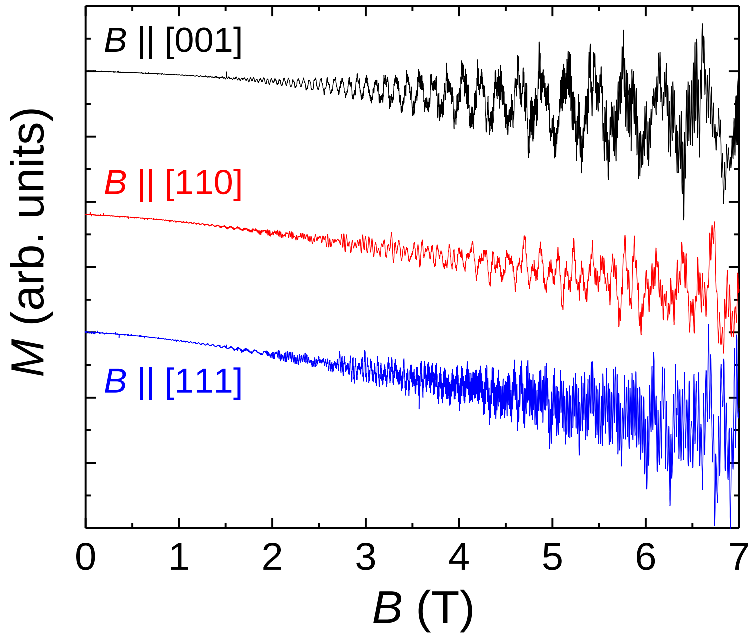

Supplementary Figure 5 | de Haas–van Alphen quantum oscillations of PtGa. Magnetization with superimposed quantum oscillations of sample S-1 with magnetic field applied along different directions—[001], [110], and [111].

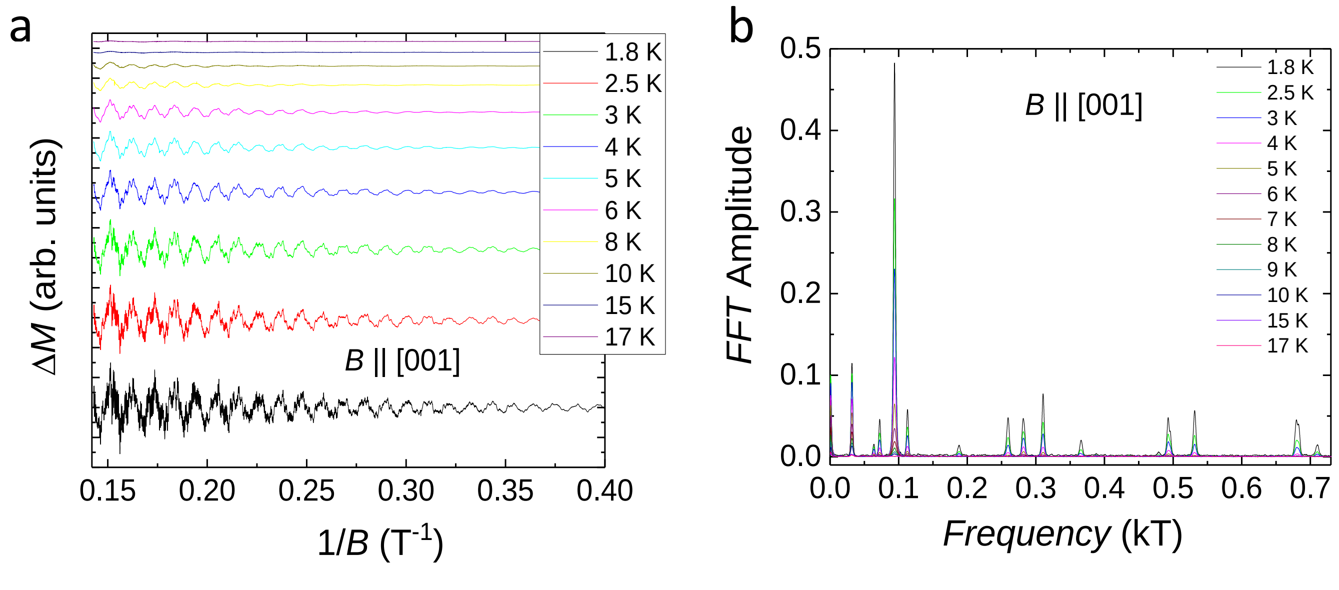

Supplementary Figure 6 | Temperature dependence of (a) dHvA quantum oscillations in PtGa and (b) FFT spectra with *B* applied along the [001] direction. The corresponding inset shows a magnified high-frequency zone.

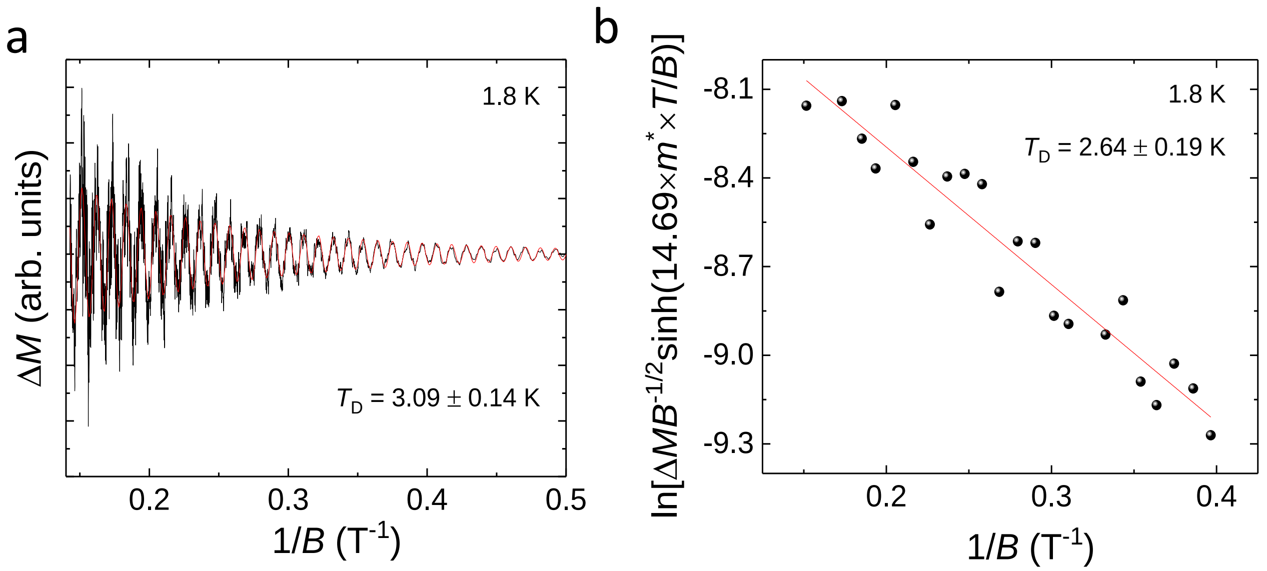

Supplementary Figure 7 | Dingle temperature of PtGa. (a) dHvA oscillations with fit using the Lifshitz–Kosevich formula for the dHvA frequency α_2 = 93.96 T while B || [001]; (b) Dingle plot of the FFT amplitude for the dHvA frequency α_2 = 93.96 T by choosing overlapping field windows with constant width in 1/B. The Dingle temperature (T_D) estimated from both methods demonstrates good agreement.

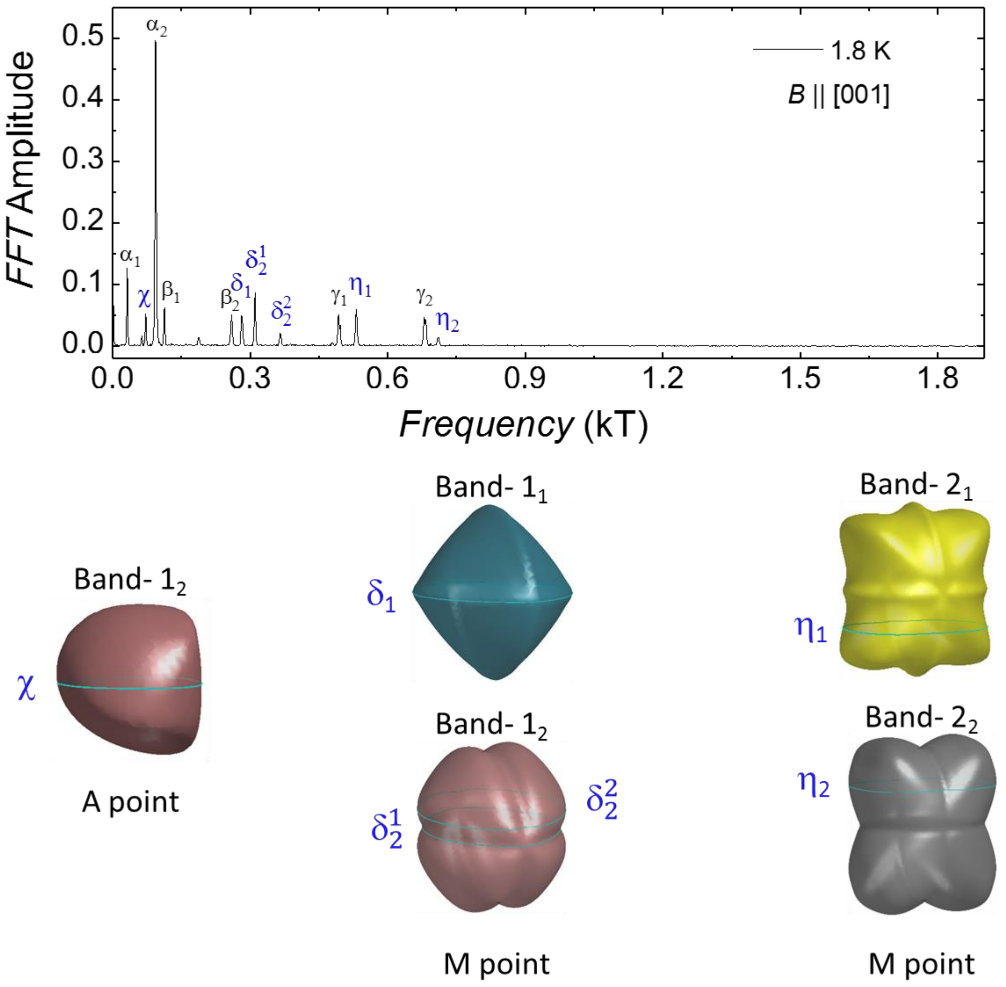

Supplementary Figure 8 | Additional spin-split Fermi pockets with identified extremal areas at different points of the Brillouin zone for different bands as shown in Fig. S-4. The corresponding frequencies are indicated in the FFT spectrum of the dHvA oscillations in the upper panel.

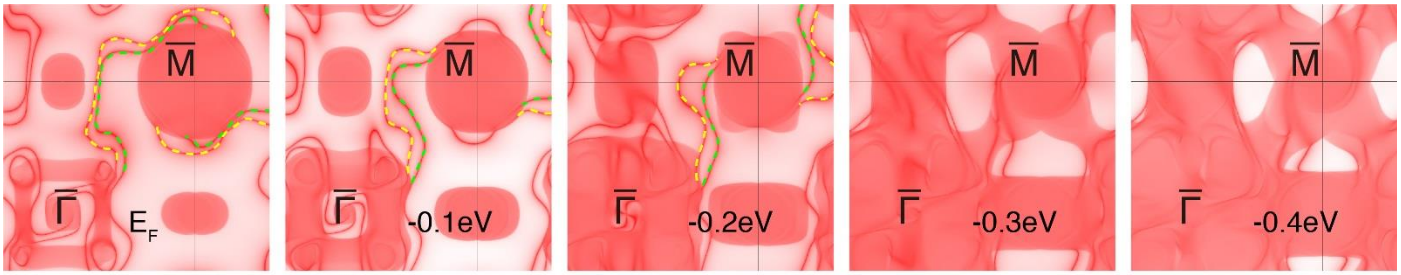

Supplementary Figure 9 | Calculated constant energy maps with different binding energies, $\Delta E = E\text{-}E_F$ = -0.1, -0.2, -0.3, and -0.4 eV, respectively. The Fermi arcs are highlighted by yellow and green dashed lines.

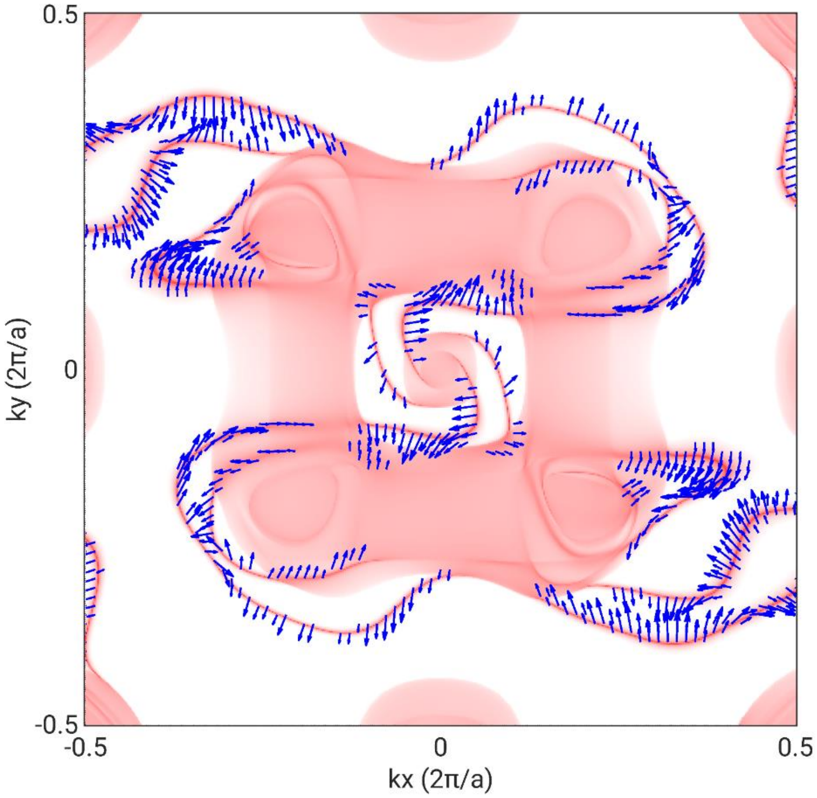

Supplementary Figure 10 | Spin-split Fermi arc of PtGa. Calculated spin texture of the surface state at $\Delta E = E - E_F$ = -0.1 eV.

Supplementary Table 1 | Room-temperature single-crystal refinement result for PtGa.

Compound	PtGa
F.W. (g/mol);	264.81
Space group; Z	$P2_13$ (No.198); 4
a (Å)	4.9114(3)
V (Å^3)	118.47(2)
Absorption Correction	Multi-scan
Extinction Coefficient	0.015(2)
θ range (deg)	5.9-35.7
No. reflections; R_{int}	1252; 0.0320
No. independent reflections	188
No. parameters	8
R_1; wR_2 (all I)	0.0213; 0.0430
Goodness of fit	1.082
Fourier difference peak and hole (e^-/Å^3)	2.432; -1.764

Supplementary Table 2 | Oscillation frequency F, effective mass m^*, extremal cross-sectional areas A_F, Fermi wave vector k_F and Fermi velocity v_F of the Fermi-surface pockets as depicted in the inset of Fig. 2(d). The analysis is performed by describing the temperature-dependent FFT amplitudes of the corresponding frequencies with the Lifshitz–Kosevich formula and the Onsager relations given in the main text.

	F (T)	m^*/m_0	A_F (Å^{-2})	k_F (Å^{-1})	v_F (m/s) (× 10^5)
α_1	31.9	0.06	0.003	0.03	7.21
α_2	93.96	0.12	0.009	0.053	5.33
β_1	112.84	0.13	0.011	0.058	5.42
β_2	259.4	0.17	0.025	0.089	6.05
γ_1	492.6	0.14	0.047	0.122	9.84
γ_2	679.37	0.19	0.065	0.144	8.76

Supplementary Note 1| Structural characterization of PtGa single crystal:

The single crystal diffraction data were collected on a Rigaku AFC7 four-circle diffractometer with a Saturn 724+ CCD-detector applying graphite-monochromatized Mo-Kα radiation. Small crystal pieces were broken from the big chunk and the fragments were mounted on Kapton loops for the data collection. The final refinement details are given in Table-S1. The complete crystallographic information has been deposited and is available citing CSD-1991900.
